# μ-Acetato-μ-(5-chloro-2-{1,3-bis[2-(5-chloro-2-oxidobenzylideneamino)ethyl]imidazolidin-2-yl}phenolato)-bis[methanolnickel(II)] methanol monosolvate monohydrate

**DOI:** 10.1107/S1600536811032727

**Published:** 2011-08-17

**Authors:** Ahmed Raza Khan, Yohannes Tesema, Ray J. Butcher, Yilma Gultneh

**Affiliations:** a Department of Chemistry, Howard University, 525 College Street NW, Washington DC 20059, USA

## Abstract

The crystal structure shows that the title compound, [Ni_2_(CH_3_CO_2_)(C_27_H_24_Cl_3_N_4_O_3_)(CH_4_O)_2_]·CH_3_OH·H_2_O, con­tains [Ni_2_ *L*(OAc)(CH_3_OH)_2_] mol­ecules in the unit cell {H_3_ *L* = 5-chloro-2-{1,3-bis[2-(5-chloro-2-oxidobenzylideneimino)-ethyl]imidazolidin-2-yl}phenolate} with water and methanol as solvates. The title compound is a neutral dinuclear compound, in which the *L* ^3−^ Schiff base acts as a hepta­dentate ligand, using each one of its N_2_O compartments to coordinate a nickel atom. The acetate anion bridges the two nickel atoms *via* one O while the distorted octahedral coordination sphere for each nickel atom is completed by a coordinated methanol ligand. One of the coordinated methanol ligands is involved in an intra­molecular hydrogen bond to the uncoordinated O atom of the bridging acetate ligand while the other forms a hydrogen bond with the methanol solvate. The solvate water mol­ecule forms strong hydrogen bonds to both terminal phenolato O atoms. The methanol solvate mol­ecule also forms a hydrogen bond with the water solvate mol­ecule.

## Related literature

For dinuclear nickel compounds containing ligands with a predefined ground state, see: Fondo *et al.* (2005 ▶, 2007 ▶, 2009 ▶); Fondo, Garcia-Deibe *et al.* (2006 ▶); Fondo, Ocampo *et al.* (2006 ▶); Lu *et al.* (2007 ▶); Paital *et al.* (2007 ▶, 2009 ▶). For density functional theory (DFT) calculations, see: Fondo *et al.* (2005 ▶).
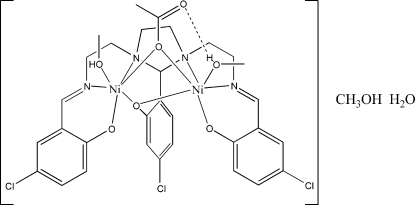


## Experimental

### 

#### Crystal data

[Ni_2_(C_2_H_3_O_2_)(C_27_H_24_Cl_3_N_4_O_3_)(CH_4_O)_2_]·CH_4_O·H_2_O
*M* *_r_* = 849.46
Orthorhombic, 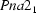

*a* = 16.684 (2) Å
*b* = 16.042 (2) Å
*c* = 13.7868 (19) Å
*V* = 3690.1 (9) Å^3^
*Z* = 4
Mo *K*α radiation
μ = 1.29 mm^−1^
*T* = 173 K
0.45 × 0.40 × 0.20 mm

#### Data collection

Bruker SMART 1000 CCD area-detector diffractometer
Absorption correction: multi-scan (*SADABS*; Sheldrick, 2003 ▶) *T*_min_ = 0.685, *T*_max_ = 1.000
23326 measured reflections
8737 independent reflections
7189 reflections with *I* > 2σ(*I*)
*R*_int_ = 0.037

#### Refinement

*R*[*F* ^2^ > 2σ(*F* ^2^)] = 0.033
*wR*(*F* ^2^) = 0.064
*S* = 0.97
8737 reflections
464 parameters
4 restraints
H atoms treated by a mixture of independent and constrained refinement
Δρ_max_ = 0.34 e Å^−3^
Δρ_min_ = −0.23 e Å^−3^
Absolute structure: Flack (1983 ▶), 3935 Friedel pairs
Flack parameter: 0.017 (8)

### 

Data collection: *SMART* (Bruker, 2000 ▶); cell refinement: *SMART*; data reduction: *SAINT-Plus* (Bruker, 2000 ▶); program(s) used to solve structure: *SHELXS97* (Sheldrick, 2008 ▶); program(s) used to refine structure: *SHELXL97* (Sheldrick, 2008 ▶); molecular graphics: *SHELXTL* (Sheldrick, 2008 ▶); software used to prepare material for publication: *SHELXTL*.

## Supplementary Material

Crystal structure: contains datablock(s) I, global. DOI: 10.1107/S1600536811032727/jj2099sup1.cif

Structure factors: contains datablock(s) I. DOI: 10.1107/S1600536811032727/jj2099Isup2.hkl

Additional supplementary materials:  crystallographic information; 3D view; checkCIF report

## Figures and Tables

**Table 1:** Hydrogen-bond geometry (Å, °)

*D*—H⋯*A*	*D*—H	H⋯*A*	*D*⋯*A*	*D*—H⋯*A*
O1*MA*—H1*MK*⋯O2*AA*	0.84	1.77	2.586 (3)	162
O1*MA*—H1*MK*⋯O1*AA*	0.84	2.66	3.029 (2)	108
O1*W*—H1*W*1⋯O1*A*	0.82 (2)	1.86 (2)	2.679 (3)	175 (4)
O1*W*—H1*W*2⋯O1*B*	0.81 (2)	1.91 (2)	2.708 (3)	174 (3)
O1*M*—H1*M*⋯O1*W*^i^	0.84	1.74	2.577 (3)	170
O1*MB*—H1*MJ*⋯O1*M*	0.84	1.83	2.658 (3)	167

Symmetry codes: (i) 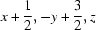; (ii) 
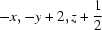
.

**Fig. 1.**
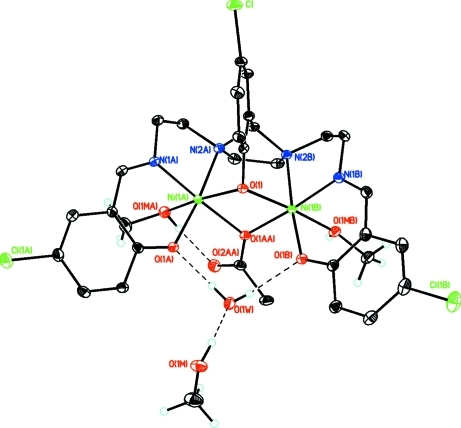
Diagram of C32H41Cl3N4Ni2O9, showing atom labeling. All H atoms except those attached to water, methanol and acetate are removed for clarity. Hydrogen bonds are shown by dashed lines.

**Fig. 2.**
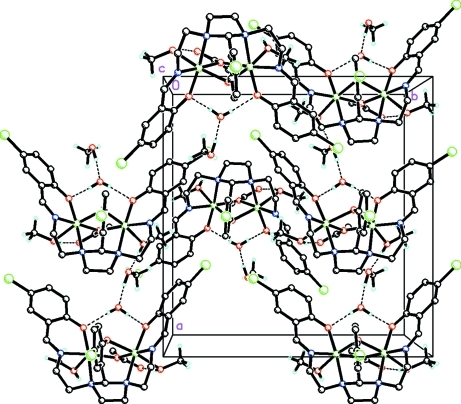
The molecular packing for C32H41Cl3N4Ni2O9 viewed down the c axis. Hydrogen bonds are shown by dashed lines.

## supplementary crystallographic information

### Comment

Nickel complexes of the compartmental triprotic heptadentate ligand, 2-hydroxyphenyl)-1,3-bis[4-(2- hydroxyphenyl)-3-azabut- 3-enyl]-1,3-imidazolidine and its derivatives have been of interest for their ability to give rise to dinuclear compounds with a predefined ground state (Fondo *et al.*, 2005, 2007, 2009; Fondo, Garcia-Deibe *et al.*, 2006; Fondo, Ocampo *et al.*, 2006; Lu *et al.*, 2007; Paital, *et al.*, 2007, 2009). Density functional theory (DFT) calculations demonstrated that the Schiff base provides an NCN bridge between the metal ions that helps to mediate the ferromagnetic exchange (Fondo, *et al.*, 2005). Consequently, the use of suitable cross-linking ligands between the dinuclear units could be a route to produce complexes of higher nuclearity, with all of the unpaired electrons aligned parallel to each other. The type of complex obtained depends on the synthesis conditions as the coordination environment about the metals is usually completed by coordinating solvent molecules.

The crystal structure shows that C_32_H_41_Cl_3_N_4_Ni_2_O_9_, (I), contains [Ni_2_L(OAc)(CH_3_OH)_2_] molecules in the unit cell (H_3_L = 2-(5-chloro-2-hydroxyphenyl)-1,3-bis[4-(5-chloro-2- hydroxyphenyl)-3-azabut-3-enyl]-1,3-imidazolidine) with water and methanol as solvates. (I) is a neutral dinuclear compound, where the *L*^3-^ Schiff base acts as a compartmental trianionic heptadentate ligand, using each one of its N_2_O compartments to coordinate a nickel atom. Thus, the metal atoms are joined to one terminal phenol oxygen (O1A, O1B), an iminic nitrogen (N1A, N1B), and an aminic nitrogen atom (N2A, N2B), with the aminic NCN group (N2A—C7—N2B) acting as a bridge between both nickel ions. In addition, the nickel centers are linked by the endogenous phenolate oxygen atom (O1) of the central ligand arm and by an exogenous bridging monodentate acetate group (O1AA). This gives rise to a nearly planar Ni_2_O_2_ metallacycle, with an intramolecular Ni—Ni distance of 3.1078 (6) Å. The coordination spheres of the nickel atoms are completed by methanol molecules. Therefore, the metal centers are hexacoordinated in a N_2_O_4_ environment, with an octahedral geometry. The Ni—O and Ni—N distances, as well as the angles about the metal atoms, show quite regular polyhedra around the central ions, with both the Ni—O_phenol_—Ni and Ni—O_acetate_—Ni angles being similar [97.98 (7)° and 97.37 (8)°, respectively]. There are similar structures reported in the literature which differ only in the nature of the coordinating solvent (H_2_O) and solvate molecules (H_2_O, CH_3_CN) in the lattice (Fondo, Ocampo *et al.*, 2006).

One of the coordinated methanol ligands is involved in an intramolecular hydrogen bond to the uncoordinated O atom (O2AA) of the bridging acetate ligand while the other forms a hydrogen bond with the methanol solvate. The solvate water molecule forms strong hydrogen bonds to both O1A and O1B. The methanol solvate molecule also forms a hydrogen bond with the water solvate molecule.

### Experimental

For the synthesis of the ligand (H_3_L) methanol solutions of triethylenetetramine and 5-chlorosalicylaldehyde were mixed in 1:3 mol ratio. After heating at 60° C for a few minutes, ether was added to this mixture, and the yellow crystals were separated, filtered and recrystallized from methanol solution. Mp 103–104° C. For synthesis of the complex, to a stirred methanol solution (25 ml) of [Ni(O_2_CCH_3_)_2_]4H_2_O (1.5 g, 2.67 mmol) was added 1.33 g (5.35 mmol) of the ligand H_3_L. Slow evaporation of the green filtrate overnight yielded green cystals suitable for X-ray analysis in 75% yield.

### Refinement

H atoms were placed in geometrically idealized positions and constrained to ride on their parent atoms with an O—H distance of 0.84 Å and C—H distances of 0.95 - 0.99 Å [*U*_iso_(H) = 1.2*U*_eq_(OH, CH, CH~2~) [*U*_iso_(H) = 1.5*U*_eq_(CH_3_)]. Water H atoms were refined isotropically with O—H distances restrained to 0.82 Å and H—O—H angle to 104.5° with [*U*_iso_(H) = 1.5*U*_eq_(O)].

### Crystal data

**Table:**

[Ni_2_(C_2_H_3_O_2_)(CH_4_O)_2_(C_27_H_24_Cl_3_N_4_O_3_)]·CH_4_O·H_2_O	*F*(000) = 1760
*M**_r_* = 849.46	*D*_x_ = 1.529 Mg m^−^^3^
Orthorhombic, *P**n**a*2_1_	Mo *K*α radiation, λ = 0.71073 Å
Hall symbol: P 2c -2n	Cell parameters from 4340 reflections
*a* = 16.684 (2) Å	θ = 2.3–27.1°
*b* = 16.042 (2) Å	µ = 1.29 mm^−^^1^
*c* = 13.7868 (19) Å	*T* = 173 K
*V* = 3690.1 (9) Å^3^	Chunk, green
*Z* = 4	0.45 × 0.40 × 0.20 mm

### Data collection

**Table:**

Bruker SMART 1000 CCD area detector diffractometer	8737 independent reflections
Radiation source: fine-focus sealed tube	7189 reflections with *I* > 2σ(*I*)
graphite	*R*_int_ = 0.037
φ and ω scans	θ_max_ = 28.4°, θ_min_ = 1.8°
Absorption correction: multi-scan (*SADABS*; Sheldrick, 2003)	*h* = −15→22
*T*_min_ = 0.685, *T*_max_ = 1.000	*k* = −18→21
23326 measured reflections	*l* = −18→18

### Refinement

**Table:**

Refinement on *F*^2^	Secondary atom site location: difference Fourier map
Least-squares matrix: full	Hydrogen site location: inferred from neighbouring sites
*R*[*F*^2^ > 2σ(*F*^2^)] = 0.033	H atoms treated by a mixture of independent and constrained refinement
*wR*(*F*^2^) = 0.064	*w* = 1/[σ^2^(*F*_o_^2^) + (0.0269*P*)^2^] where *P* = (*F*_o_^2^ + 2*F*_c_^2^)/3
*S* = 0.97	(Δ/σ)_max_ = 0.001
8737 reflections	Δρ_max_ = 0.34 e Å^−^^3^
464 parameters	Δρ_min_ = −0.23 e Å^−^^3^
4 restraints	Absolute structure: Flack (1983), no. of Friedel pairs?
Primary atom site location: structure-invariant direct methods	Flack parameter: 0.017 (8)

### Special details

**Table:**

Geometry. All e.s.d.'s (except the e.s.d. in the dihedral angle between two l.s. planes) are estimated using the full covariance matrix. The cell e.s.d.'s are taken into account individually in the estimation of e.s.d.'s in distances, angles and torsion angles; correlations between e.s.d.'s in cell parameters are only used when they are defined by crystal symmetry. An approximate (isotropic) treatment of cell e.s.d.'s is used for estimating e.s.d.'s involving l.s. planes.
Refinement. Refinement of *F*^2^ against ALL reflections. The weighted *R*-factor *wR* and goodness of fit *S* are based on *F*^2^, conventional *R*-factors *R* are based on *F*, with *F* set to zero for negative *F*^2^. The threshold expression of *F*^2^ > σ(*F*^2^) is used only for calculating *R*-factors(gt) *etc*. and is not relevant to the choice of reflections for refinement. *R*-factors based on *F*^2^ are statistically about twice as large as those based on *F*, and *R*- factors based on ALL data will be even larger.

### Fractional atomic coordinates and isotropic or equivalent isotropic displacement parameters (Å^2^)

**Table:**

	*x*	*y*	*z*	*U*_iso_*/*U*_eq_	
Ni1A	0.030884 (18)	0.86755 (2)	0.49661 (3)	0.02591 (8)	
Ni1B	0.038838 (18)	0.67711 (2)	0.45640 (2)	0.02566 (8)	
Cl	−0.00326 (4)	0.73145 (5)	0.96738 (6)	0.04296 (17)	
Cl1A	−0.31065 (5)	1.15093 (5)	0.53792 (6)	0.0493 (2)	
Cl1B	−0.25128 (5)	0.34351 (5)	0.37379 (8)	0.0560 (2)	
O1	−0.02255 (10)	0.75823 (11)	0.54367 (13)	0.0258 (4)	
O1A	−0.06659 (10)	0.90623 (11)	0.42579 (13)	0.0305 (4)	
O1B	−0.05824 (11)	0.64873 (11)	0.37941 (14)	0.0308 (4)	
O1AA	0.05959 (11)	0.78731 (10)	0.38367 (13)	0.0280 (4)	
O2AA	0.10906 (13)	0.87725 (13)	0.27867 (15)	0.0471 (5)	
O1MA	0.09955 (10)	0.96620 (11)	0.43472 (14)	0.0371 (5)	
H1MK	0.1099	0.9446	0.3806	0.044*	
O1MB	0.11323 (11)	0.60759 (11)	0.35587 (14)	0.0344 (4)	
H1MJ	0.1542	0.6274	0.3290	0.041*	
O1W	−0.13355 (13)	0.78777 (13)	0.31538 (18)	0.0484 (6)	
H1W1	−0.1106 (19)	0.8238 (14)	0.347 (3)	0.073*	
H1W2	−0.113 (2)	0.7444 (12)	0.332 (3)	0.073*	
O1M	0.22768 (12)	0.67412 (14)	0.24637 (16)	0.0470 (5)	
H1M	0.2703	0.6909	0.2723	0.056*	
N1A	0.00600 (13)	0.94058 (13)	0.60946 (16)	0.0295 (5)	
N2A	0.13725 (12)	0.83765 (13)	0.57830 (16)	0.0278 (5)	
N1B	0.02800 (13)	0.57248 (14)	0.53500 (17)	0.0309 (5)	
N2B	0.14429 (12)	0.69703 (13)	0.54912 (15)	0.0261 (5)	
C1	−0.01809 (15)	0.74633 (16)	0.63923 (19)	0.0256 (6)	
C2	−0.08666 (16)	0.73573 (16)	0.6957 (2)	0.0311 (6)	
H2A	−0.1376	0.7333	0.6650	0.037*	
C3	−0.08225 (16)	0.72863 (17)	0.7957 (2)	0.0341 (7)	
H3A	−0.1296	0.7217	0.8330	0.041*	
C4	−0.00883 (18)	0.73162 (17)	0.84032 (19)	0.0319 (6)	
C5	0.06045 (16)	0.73903 (17)	0.7878 (2)	0.0302 (6)	
H5A	0.1109	0.7397	0.8198	0.036*	
C6	0.05649 (16)	0.74559 (17)	0.6871 (2)	0.0272 (6)	
C7	0.13219 (15)	0.75677 (16)	0.63004 (19)	0.0284 (6)	
H7A	0.1788	0.7524	0.6754	0.034*	
C8	0.21070 (15)	0.82688 (16)	0.5174 (2)	0.0346 (7)	
H8A	0.2591	0.8455	0.5529	0.041*	
H8B	0.2063	0.8590	0.4564	0.041*	
C9	0.21425 (15)	0.73351 (16)	0.4968 (2)	0.0341 (6)	
H9A	0.2102	0.7227	0.4263	0.041*	
H9B	0.2651	0.7094	0.5211	0.041*	
C1A	−0.11614 (15)	1.00242 (15)	0.5459 (2)	0.0283 (6)	
C2A	−0.11907 (14)	0.96151 (15)	0.4553 (2)	0.0268 (5)	
C3A	−0.18261 (15)	0.98341 (15)	0.3918 (2)	0.0298 (6)	
H3AA	−0.1859	0.9572	0.3302	0.036*	
C4A	−0.23901 (15)	1.04105 (16)	0.4167 (2)	0.0327 (6)	
H4AA	−0.2806	1.0548	0.3724	0.039*	
C5A	−0.23586 (15)	1.07982 (15)	0.5068 (2)	0.0336 (6)	
C6A	−0.17552 (16)	1.06147 (16)	0.5699 (2)	0.0346 (7)	
H6AA	−0.1734	1.0889	0.6309	0.041*	
C7A	−0.05340 (16)	0.99102 (17)	0.6167 (2)	0.0330 (6)	
H7AA	−0.0563	1.0239	0.6740	0.040*	
C8A	0.06633 (17)	0.93926 (18)	0.6871 (2)	0.0359 (7)	
H8AA	0.0496	0.9004	0.7391	0.043*	
H8AB	0.0722	0.9956	0.7155	0.043*	
C9A	0.14602 (16)	0.91075 (17)	0.6434 (2)	0.0360 (7)	
H9AA	0.1698	0.9575	0.6063	0.043*	
H9AB	0.1834	0.8964	0.6966	0.043*	
C1B	−0.08523 (15)	0.51394 (16)	0.4483 (2)	0.0331 (6)	
C2B	−0.10109 (15)	0.58068 (16)	0.3839 (2)	0.0283 (6)	
C3B	−0.16738 (16)	0.57244 (18)	0.3199 (2)	0.0357 (7)	
H3BA	−0.1803	0.6171	0.2774	0.043*	
C4B	−0.21304 (17)	0.50132 (17)	0.3181 (2)	0.0380 (7)	
H4BA	−0.2572	0.4972	0.2749	0.046*	
C5B	−0.19473 (17)	0.43556 (17)	0.3792 (2)	0.0399 (7)	
C6B	−0.13271 (16)	0.44085 (17)	0.4433 (2)	0.0383 (7)	
H6BA	−0.1212	0.3952	0.4849	0.046*	
C7B	−0.02163 (16)	0.51340 (17)	0.5188 (2)	0.0341 (7)	
H7BA	−0.0160	0.4645	0.5571	0.041*	
C8B	0.08865 (17)	0.56281 (17)	0.6114 (2)	0.0350 (7)	
H8BA	0.1020	0.5031	0.6198	0.042*	
H8BB	0.0676	0.5842	0.6737	0.042*	
C9B	0.16279 (17)	0.61102 (17)	0.5827 (2)	0.0352 (7)	
H9BA	0.1995	0.6141	0.6390	0.042*	
H9BB	0.1908	0.5807	0.5302	0.042*	
C1AA	0.07482 (17)	0.81086 (19)	0.2963 (2)	0.0342 (7)	
C2AA	0.04755 (19)	0.7573 (2)	0.2136 (2)	0.0423 (8)	
H2AA	0.0918	0.7499	0.1676	0.064*	
H2AB	0.0023	0.7841	0.1808	0.064*	
H2AC	0.0307	0.7028	0.2384	0.064*	
C1M	0.2436 (2)	0.6485 (2)	0.1504 (3)	0.0623 (10)	
H1M1	0.3016	0.6431	0.1412	0.093*	
H1M2	0.2224	0.6900	0.1050	0.093*	
H1M3	0.2178	0.5946	0.1383	0.093*	
C1MA	0.06774 (19)	1.04743 (19)	0.4206 (3)	0.0523 (9)	
H1MA	0.0979	1.0758	0.3692	0.078*	
H1MB	0.0721	1.0793	0.4810	0.078*	
H1MC	0.0112	1.0433	0.4017	0.078*	
C1MB	0.08373 (18)	0.53940 (19)	0.3000 (2)	0.0424 (8)	
H1MD	0.1270	0.5171	0.2595	0.064*	
H1ME	0.0397	0.5585	0.2586	0.064*	
H1MF	0.0642	0.4956	0.3436	0.064*	

### Atomic displacement parameters (Å^2^)

**Table:**

	*U*^11^	*U*^22^	*U*^33^	*U*^12^	*U*^13^	*U*^23^
Ni1A	0.02723 (16)	0.02880 (17)	0.02170 (16)	0.00058 (14)	−0.00341 (15)	−0.00147 (15)
Ni1B	0.02737 (16)	0.02862 (16)	0.02098 (16)	0.00225 (14)	−0.00269 (16)	−0.00049 (15)
Cl	0.0478 (4)	0.0595 (4)	0.0215 (3)	0.0089 (4)	0.0037 (3)	0.0022 (3)
Cl1A	0.0481 (4)	0.0472 (4)	0.0526 (5)	0.0197 (4)	−0.0015 (4)	−0.0020 (4)
Cl1B	0.0358 (4)	0.0433 (4)	0.0888 (7)	−0.0108 (4)	0.0096 (4)	−0.0133 (4)
O1	0.0267 (10)	0.0302 (9)	0.0206 (9)	0.0003 (8)	−0.0029 (8)	0.0008 (8)
O1A	0.0322 (10)	0.0323 (10)	0.0270 (11)	0.0054 (8)	−0.0074 (8)	−0.0037 (8)
O1B	0.0334 (10)	0.0295 (9)	0.0294 (11)	−0.0008 (8)	−0.0069 (8)	−0.0003 (8)
O1AA	0.0337 (10)	0.0306 (9)	0.0196 (10)	−0.0001 (8)	0.0003 (8)	0.0006 (8)
O2AA	0.0664 (15)	0.0415 (12)	0.0334 (12)	−0.0027 (11)	0.0092 (11)	0.0108 (10)
O1MA	0.0406 (11)	0.0353 (11)	0.0353 (13)	−0.0030 (9)	0.0002 (9)	0.0039 (9)
O1MB	0.0335 (11)	0.0397 (10)	0.0300 (11)	0.0022 (9)	−0.0006 (9)	−0.0076 (9)
O1W	0.0473 (13)	0.0372 (12)	0.0608 (16)	0.0120 (10)	−0.0267 (11)	−0.0160 (11)
O1M	0.0363 (12)	0.0700 (15)	0.0346 (13)	−0.0094 (11)	−0.0052 (10)	−0.0008 (11)
N1A	0.0333 (12)	0.0296 (12)	0.0255 (13)	0.0019 (10)	−0.0059 (10)	−0.0050 (10)
N2A	0.0247 (11)	0.0330 (12)	0.0257 (12)	−0.0012 (10)	−0.0023 (9)	−0.0010 (10)
N1B	0.0338 (12)	0.0326 (12)	0.0263 (12)	0.0042 (10)	−0.0010 (10)	−0.0002 (10)
N2B	0.0255 (11)	0.0323 (11)	0.0206 (12)	0.0051 (9)	−0.0001 (9)	−0.0010 (9)
C1	0.0293 (14)	0.0247 (12)	0.0227 (14)	0.0017 (11)	−0.0012 (11)	−0.0026 (11)
C2	0.0287 (15)	0.0358 (15)	0.0289 (16)	0.0010 (12)	−0.0007 (12)	0.0024 (12)
C3	0.0290 (15)	0.0413 (16)	0.0320 (17)	0.0020 (12)	0.0042 (13)	0.0041 (13)
C4	0.0412 (16)	0.0365 (16)	0.0181 (14)	0.0091 (13)	0.0007 (12)	0.0001 (11)
C5	0.0280 (14)	0.0402 (16)	0.0223 (14)	0.0040 (12)	−0.0031 (12)	−0.0012 (12)
C6	0.0281 (14)	0.0307 (14)	0.0228 (14)	0.0042 (12)	−0.0011 (11)	−0.0021 (12)
C7	0.0263 (14)	0.0372 (15)	0.0217 (14)	0.0022 (12)	−0.0042 (11)	−0.0019 (12)
C8	0.0244 (13)	0.0456 (16)	0.0337 (18)	−0.0011 (12)	0.0012 (12)	0.0048 (13)
C9	0.0237 (13)	0.0536 (18)	0.0249 (14)	0.0019 (12)	0.0024 (12)	−0.0007 (14)
C1A	0.0302 (14)	0.0252 (13)	0.0295 (15)	0.0012 (11)	−0.0041 (12)	−0.0014 (12)
C2A	0.0269 (13)	0.0256 (12)	0.0280 (13)	−0.0022 (10)	−0.0035 (12)	0.0038 (12)
C3A	0.0312 (14)	0.0328 (14)	0.0254 (15)	−0.0012 (12)	−0.0029 (12)	0.0011 (12)
C4A	0.0246 (14)	0.0362 (15)	0.0374 (17)	−0.0013 (12)	−0.0065 (12)	0.0085 (13)
C5A	0.0324 (14)	0.0286 (14)	0.0397 (18)	0.0040 (11)	−0.0002 (14)	−0.0011 (13)
C6A	0.0406 (16)	0.0329 (15)	0.0303 (17)	0.0025 (13)	−0.0007 (13)	−0.0050 (12)
C7A	0.0401 (16)	0.0323 (14)	0.0265 (15)	0.0009 (13)	−0.0044 (13)	−0.0066 (12)
C8A	0.0397 (16)	0.0371 (16)	0.0308 (16)	0.0035 (13)	−0.0122 (13)	−0.0061 (13)
C9A	0.0357 (15)	0.0368 (16)	0.0355 (18)	−0.0012 (13)	−0.0120 (13)	−0.0046 (13)
C1B	0.0309 (14)	0.0307 (14)	0.0376 (17)	0.0024 (11)	0.0047 (13)	−0.0024 (13)
C2B	0.0262 (13)	0.0328 (14)	0.0260 (15)	0.0024 (11)	0.0038 (11)	−0.0063 (12)
C3B	0.0318 (15)	0.0375 (15)	0.0379 (18)	0.0038 (13)	−0.0021 (13)	−0.0084 (13)
C4B	0.0279 (15)	0.0436 (17)	0.0425 (19)	−0.0006 (13)	0.0034 (13)	−0.0130 (14)
C5B	0.0275 (15)	0.0362 (16)	0.056 (2)	−0.0036 (12)	0.0103 (15)	−0.0120 (15)
C6B	0.0369 (15)	0.0334 (15)	0.045 (2)	0.0000 (12)	0.0084 (15)	−0.0007 (14)
C7B	0.0404 (17)	0.0310 (14)	0.0310 (17)	0.0032 (13)	0.0023 (12)	0.0020 (12)
C8B	0.0413 (16)	0.0318 (15)	0.0318 (17)	0.0087 (13)	−0.0070 (14)	0.0043 (13)
C9B	0.0332 (16)	0.0406 (16)	0.0318 (16)	0.0087 (13)	−0.0066 (12)	−0.0021 (13)
C1AA	0.0337 (15)	0.0454 (17)	0.0234 (15)	0.0116 (13)	0.0003 (13)	0.0016 (13)
C2AA	0.0435 (19)	0.060 (2)	0.0235 (16)	0.0090 (16)	−0.0042 (13)	−0.0028 (15)
C1M	0.063 (2)	0.084 (3)	0.040 (2)	−0.012 (2)	0.0005 (18)	−0.007 (2)
C1MA	0.0531 (19)	0.0359 (17)	0.068 (3)	−0.0072 (16)	0.0005 (18)	0.0132 (16)
C1MB	0.0385 (17)	0.0477 (18)	0.0409 (19)	0.0031 (14)	−0.0020 (14)	−0.0153 (15)

### Geometric parameters (Å, °)

**Table:**

Ni1A—N1A	1.991 (2)	C8—H8A	0.9900
Ni1A—O1A	1.9957 (17)	C8—H8B	0.9900
Ni1A—O1	2.0716 (18)	C9—H9A	0.9900
Ni1A—O1AA	2.0762 (18)	C9—H9B	0.9900
Ni1A—O1MA	2.1318 (18)	C1A—C6A	1.410 (4)
Ni1A—N2A	2.156 (2)	C1A—C2A	1.412 (4)
Ni1B—O1B	1.9893 (18)	C1A—C7A	1.443 (4)
Ni1B—N1B	2.006 (2)	C2A—C3A	1.419 (3)
Ni1B—O1	2.0470 (18)	C3A—C4A	1.363 (3)
Ni1B—O1AA	2.0616 (17)	C3A—H3AA	0.9500
Ni1B—O1MB	2.1692 (19)	C4A—C5A	1.390 (4)
Ni1B—N2B	2.198 (2)	C4A—H4AA	0.9500
Cl—C4	1.754 (3)	C5A—C6A	1.362 (4)
Cl1A—C5A	1.744 (3)	C6A—H6AA	0.9500
Cl1B—C5B	1.754 (3)	C7A—H7AA	0.9500
O1—C1	1.333 (3)	C8A—C9A	1.530 (4)
O1A—C2A	1.311 (3)	C8A—H8AA	0.9900
O1B—C2B	1.307 (3)	C8A—H8AB	0.9900
O1AA—C1AA	1.287 (3)	C9A—H9AA	0.9900
O2AA—C1AA	1.233 (3)	C9A—H9AB	0.9900
O1MA—C1MA	1.421 (3)	C1B—C2B	1.415 (4)
O1MA—H1MK	0.8400	C1B—C6B	1.417 (4)
O1MB—C1MB	1.425 (3)	C1B—C7B	1.440 (4)
O1MB—H1MJ	0.8400	C2B—C3B	1.422 (4)
O1W—H1W1	0.817 (17)	C3B—C4B	1.372 (4)
O1W—H1W2	0.805 (17)	C3B—H3BA	0.9500
O1M—C1M	1.411 (4)	C4B—C5B	1.384 (4)
O1M—H1M	0.8400	C4B—H4BA	0.9500
N1A—C7A	1.283 (3)	C5B—C6B	1.363 (4)
N1A—C8A	1.469 (3)	C6B—H6BA	0.9500
N2A—C7	1.483 (3)	C7B—H7BA	0.9500
N2A—C9A	1.484 (3)	C8B—C9B	1.512 (4)
N2A—C8	1.495 (3)	C8B—H8BA	0.9900
N1B—C7B	1.278 (3)	C8B—H8BB	0.9900
N1B—C8B	1.469 (3)	C9B—H9BA	0.9900
N2B—C7	1.485 (3)	C9B—H9BB	0.9900
N2B—C9B	1.488 (3)	C1AA—C2AA	1.499 (4)
N2B—C9	1.492 (3)	C2AA—H2AA	0.9800
C1—C2	1.395 (4)	C2AA—H2AB	0.9800
C1—C6	1.408 (4)	C2AA—H2AC	0.9800
C2—C3	1.385 (4)	C1M—H1M1	0.9800
C2—H2A	0.9500	C1M—H1M2	0.9800
C3—C4	1.372 (4)	C1M—H1M3	0.9800
C3—H3A	0.9500	C1MA—H1MA	0.9800
C4—C5	1.369 (4)	C1MA—H1MB	0.9800
C5—C6	1.394 (4)	C1MA—H1MC	0.9800
C5—H5A	0.9500	C1MB—H1MD	0.9800
C6—C7	1.498 (4)	C1MB—H1ME	0.9800
C7—H7A	1.0000	C1MB—H1MF	0.9800
C8—C9	1.526 (4)		
			
N1A—Ni1A—O1A	91.69 (8)	N2B—C9—H9B	110.7
N1A—Ni1A—O1	99.42 (8)	C8—C9—H9B	110.7
O1A—Ni1A—O1	93.77 (7)	H9A—C9—H9B	108.8
N1A—Ni1A—O1AA	177.14 (8)	C6A—C1A—C2A	119.7 (2)
O1A—Ni1A—O1AA	90.80 (7)	C6A—C1A—C7A	115.9 (2)
O1—Ni1A—O1AA	79.01 (7)	C2A—C1A—C7A	124.4 (2)
N1A—Ni1A—O1MA	89.31 (8)	O1A—C2A—C1A	124.5 (2)
O1A—Ni1A—O1MA	90.65 (7)	O1A—C2A—C3A	118.3 (2)
O1—Ni1A—O1MA	170.08 (7)	C1A—C2A—C3A	117.2 (2)
O1AA—Ni1A—O1MA	92.07 (7)	C4A—C3A—C2A	121.9 (3)
N1A—Ni1A—N2A	83.93 (9)	C4A—C3A—H3AA	119.1
O1A—Ni1A—N2A	174.56 (8)	C2A—C3A—H3AA	119.1
O1—Ni1A—N2A	90.13 (7)	C3A—C4A—C5A	120.2 (3)
O1AA—Ni1A—N2A	93.66 (8)	C3A—C4A—H4AA	119.9
O1MA—Ni1A—N2A	86.10 (8)	C5A—C4A—H4AA	119.9
O1B—Ni1B—N1B	91.34 (8)	C6A—C5A—C4A	120.1 (2)
O1B—Ni1B—O1	92.96 (7)	C6A—C5A—Cl1A	120.9 (2)
N1B—Ni1B—O1	99.75 (8)	C4A—C5A—Cl1A	119.0 (2)
O1B—Ni1B—O1AA	94.21 (7)	C5A—C6A—C1A	121.0 (3)
N1B—Ni1B—O1AA	174.45 (9)	C5A—C6A—H6AA	119.5
O1—Ni1B—O1AA	79.91 (7)	C1A—C6A—H6AA	119.5
O1B—Ni1B—O1MB	90.42 (7)	N1A—C7A—C1A	126.0 (3)
N1B—Ni1B—O1MB	88.08 (8)	N1A—C7A—H7AA	117.0
O1—Ni1B—O1MB	171.38 (7)	C1A—C7A—H7AA	117.0
O1AA—Ni1B—O1MB	91.95 (7)	N1A—C8A—C9A	108.2 (2)
O1B—Ni1B—N2B	174.43 (8)	N1A—C8A—H8AA	110.0
N1B—Ni1B—N2B	83.09 (8)	C9A—C8A—H8AA	110.0
O1—Ni1B—N2B	88.06 (7)	N1A—C8A—H8AB	110.0
O1AA—Ni1B—N2B	91.36 (7)	C9A—C8A—H8AB	110.0
O1MB—Ni1B—N2B	89.33 (7)	H8AA—C8A—H8AB	108.4
C1—O1—Ni1B	117.51 (15)	N2A—C9A—C8A	112.9 (2)
C1—O1—Ni1A	114.00 (15)	N2A—C9A—H9AA	109.0
Ni1B—O1—Ni1A	97.98 (7)	C8A—C9A—H9AA	109.0
C2A—O1A—Ni1A	127.06 (17)	N2A—C9A—H9AB	109.0
C2B—O1B—Ni1B	127.68 (17)	C8A—C9A—H9AB	109.0
C1AA—O1AA—Ni1B	137.71 (18)	H9AA—C9A—H9AB	107.8
C1AA—O1AA—Ni1A	124.40 (17)	C2B—C1B—C6B	119.4 (3)
Ni1B—O1AA—Ni1A	97.37 (8)	C2B—C1B—C7B	124.5 (2)
C1MA—O1MA—Ni1A	122.36 (17)	C6B—C1B—C7B	116.1 (3)
C1MA—O1MA—H1MK	109.5	O1B—C2B—C1B	124.0 (2)
Ni1A—O1MA—H1MK	99.2	O1B—C2B—C3B	118.3 (2)
C1MB—O1MB—Ni1B	122.82 (16)	C1B—C2B—C3B	117.7 (2)
C1MB—O1MB—H1MJ	109.5	C4B—C3B—C2B	121.4 (3)
Ni1B—O1MB—H1MJ	123.5	C4B—C3B—H3BA	119.3
H1W1—O1W—H1W2	105 (3)	C2B—C3B—H3BA	119.3
C1M—O1M—H1M	109.5	C3B—C4B—C5B	120.0 (3)
C7A—N1A—C8A	118.8 (2)	C3B—C4B—H4BA	120.0
C7A—N1A—Ni1A	126.37 (19)	C5B—C4B—H4BA	120.0
C8A—N1A—Ni1A	114.69 (17)	C6B—C5B—C4B	121.0 (3)
C7—N2A—C9A	114.0 (2)	C6B—C5B—Cl1B	119.2 (2)
C7—N2A—C8	102.45 (19)	C4B—C5B—Cl1B	119.8 (2)
C9A—N2A—C8	110.5 (2)	C5B—C6B—C1B	120.5 (3)
C7—N2A—Ni1A	113.52 (15)	C5B—C6B—H6BA	119.8
C9A—N2A—Ni1A	102.78 (15)	C1B—C6B—H6BA	119.8
C8—N2A—Ni1A	114.02 (17)	N1B—C7B—C1B	126.2 (3)
C7B—N1B—C8B	119.5 (2)	N1B—C7B—H7BA	116.9
C7B—N1B—Ni1B	125.8 (2)	C1B—C7B—H7BA	116.9
C8B—N1B—Ni1B	114.43 (17)	N1B—C8B—C9B	108.8 (2)
C7—N2B—C9B	113.1 (2)	N1B—C8B—H8BA	109.9
C7—N2B—C9	102.51 (19)	C9B—C8B—H8BA	109.9
C9B—N2B—C9	110.6 (2)	N1B—C8B—H8BB	109.9
C7—N2B—Ni1B	114.96 (15)	C9B—C8B—H8BB	109.9
C9B—N2B—Ni1B	102.26 (16)	H8BA—C8B—H8BB	108.3
C9—N2B—Ni1B	113.71 (17)	N2B—C9B—C8B	112.7 (2)
O1—C1—C2	121.6 (2)	N2B—C9B—H9BA	109.0
O1—C1—C6	120.9 (2)	C8B—C9B—H9BA	109.0
C2—C1—C6	117.5 (2)	N2B—C9B—H9BB	109.0
C3—C2—C1	121.5 (3)	C8B—C9B—H9BB	109.0
C3—C2—H2A	119.3	H9BA—C9B—H9BB	107.8
C1—C2—H2A	119.3	O2AA—C1AA—O1AA	122.0 (3)
C4—C3—C2	119.4 (3)	O2AA—C1AA—C2AA	119.1 (3)
C4—C3—H3A	120.3	O1AA—C1AA—C2AA	118.9 (3)
C2—C3—H3A	120.3	C1AA—C2AA—H2AA	109.5
C5—C4—C3	121.3 (3)	C1AA—C2AA—H2AB	109.5
C5—C4—Cl	118.9 (2)	H2AA—C2AA—H2AB	109.5
C3—C4—Cl	119.7 (2)	C1AA—C2AA—H2AC	109.5
C4—C5—C6	119.6 (3)	H2AA—C2AA—H2AC	109.5
C4—C5—H5A	120.2	H2AB—C2AA—H2AC	109.5
C6—C5—H5A	120.2	O1M—C1M—H1M1	109.5
C5—C6—C1	120.6 (2)	O1M—C1M—H1M2	109.5
C5—C6—C7	119.5 (2)	H1M1—C1M—H1M2	109.5
C1—C6—C7	119.9 (2)	O1M—C1M—H1M3	109.5
N2A—C7—N2B	101.3 (2)	H1M1—C1M—H1M3	109.5
N2A—C7—C6	113.9 (2)	H1M2—C1M—H1M3	109.5
N2B—C7—C6	115.6 (2)	O1MA—C1MA—H1MA	109.5
N2A—C7—H7A	108.6	O1MA—C1MA—H1MB	109.5
N2B—C7—H7A	108.6	H1MA—C1MA—H1MB	109.5
C6—C7—H7A	108.6	O1MA—C1MA—H1MC	109.5
N2A—C8—C9	104.5 (2)	H1MA—C1MA—H1MC	109.5
N2A—C8—H8A	110.9	H1MB—C1MA—H1MC	109.5
C9—C8—H8A	110.9	O1MB—C1MB—H1MD	109.5
N2A—C8—H8B	110.9	O1MB—C1MB—H1ME	109.5
C9—C8—H8B	110.9	H1MD—C1MB—H1ME	109.5
H8A—C8—H8B	108.9	O1MB—C1MB—H1MF	109.5
N2B—C9—C8	105.3 (2)	H1MD—C1MB—H1MF	109.5
N2B—C9—H9A	110.7	H1ME—C1MB—H1MF	109.5
C8—C9—H9A	110.7		
			
O1B—Ni1B—O1—C1	125.63 (17)	Ni1A—O1—C1—C6	−56.3 (3)
N1B—Ni1B—O1—C1	33.76 (18)	O1—C1—C2—C3	−175.9 (3)
O1AA—Ni1B—O1—C1	−140.61 (18)	C6—C1—C2—C3	3.0 (4)
N2B—Ni1B—O1—C1	−48.88 (18)	C1—C2—C3—C4	−0.3 (4)
O1B—Ni1B—O1—Ni1A	−111.96 (8)	C2—C3—C4—C5	−2.0 (4)
N1B—Ni1B—O1—Ni1A	156.17 (8)	C2—C3—C4—Cl	174.7 (2)
O1AA—Ni1B—O1—Ni1A	−18.20 (7)	C3—C4—C5—C6	1.5 (4)
N2B—Ni1B—O1—Ni1A	73.53 (8)	Cl—C4—C5—C6	−175.2 (2)
N1A—Ni1A—O1—C1	−34.51 (17)	C4—C5—C6—C1	1.3 (4)
O1A—Ni1A—O1—C1	−126.84 (16)	C4—C5—C6—C7	177.8 (2)
O1AA—Ni1A—O1—C1	143.07 (17)	O1—C1—C6—C5	175.4 (2)
N2A—Ni1A—O1—C1	49.37 (17)	C2—C1—C6—C5	−3.4 (4)
N1A—Ni1A—O1—Ni1B	−159.46 (8)	O1—C1—C6—C7	−1.1 (4)
O1A—Ni1A—O1—Ni1B	108.20 (8)	C2—C1—C6—C7	−179.9 (2)
O1AA—Ni1A—O1—Ni1B	18.12 (7)	C9A—N2A—C7—N2B	−166.61 (19)
N2A—Ni1A—O1—Ni1B	−75.58 (8)	C8—N2A—C7—N2B	−47.3 (2)
N1A—Ni1A—O1A—C2A	1.2 (2)	Ni1A—N2A—C7—N2B	76.2 (2)
O1—Ni1A—O1A—C2A	100.7 (2)	C9A—N2A—C7—C6	68.7 (3)
O1AA—Ni1A—O1A—C2A	179.77 (19)	C8—N2A—C7—C6	−172.0 (2)
O1MA—Ni1A—O1A—C2A	−88.2 (2)	Ni1A—N2A—C7—C6	−48.6 (3)
N1B—Ni1B—O1B—C2B	−4.7 (2)	C9B—N2B—C7—N2A	165.3 (2)
O1—Ni1B—O1B—C2B	−104.5 (2)	C9—N2B—C7—N2A	46.2 (2)
O1AA—Ni1B—O1B—C2B	175.4 (2)	Ni1B—N2B—C7—N2A	−77.7 (2)
O1MB—Ni1B—O1B—C2B	83.4 (2)	C9B—N2B—C7—C6	−71.1 (3)
O1B—Ni1B—O1AA—C1AA	−61.0 (3)	C9—N2B—C7—C6	169.8 (2)
O1—Ni1B—O1AA—C1AA	−153.2 (3)	Ni1B—N2B—C7—C6	45.9 (3)
O1MB—Ni1B—O1AA—C1AA	29.6 (3)	C5—C6—C7—N2A	−115.5 (3)
N2B—Ni1B—O1AA—C1AA	119.0 (3)	C1—C6—C7—N2A	61.0 (3)
O1B—Ni1B—O1AA—Ni1A	110.40 (8)	C5—C6—C7—N2B	127.8 (3)
O1—Ni1B—O1AA—Ni1A	18.13 (7)	C1—C6—C7—N2B	−55.7 (3)
O1MB—Ni1B—O1AA—Ni1A	−159.04 (8)	C7—N2A—C8—C9	29.8 (3)
N2B—Ni1B—O1AA—Ni1A	−69.66 (8)	C9A—N2A—C8—C9	151.5 (2)
O1A—Ni1A—O1AA—C1AA	61.3 (2)	Ni1A—N2A—C8—C9	−93.3 (2)
O1—Ni1A—O1AA—C1AA	155.0 (2)	C7—N2B—C9—C8	−27.3 (3)
O1MA—Ni1A—O1AA—C1AA	−29.4 (2)	C9B—N2B—C9—C8	−148.2 (2)
N2A—Ni1A—O1AA—C1AA	−115.6 (2)	Ni1B—N2B—C9—C8	97.4 (2)
O1A—Ni1A—O1AA—Ni1B	−111.65 (8)	N2A—C8—C9—N2B	−1.5 (3)
O1—Ni1A—O1AA—Ni1B	−17.96 (7)	Ni1A—O1A—C2A—C1A	−1.7 (3)
O1MA—Ni1A—O1AA—Ni1B	157.67 (7)	Ni1A—O1A—C2A—C3A	177.05 (17)
N2A—Ni1A—O1AA—Ni1B	71.45 (8)	C6A—C1A—C2A—O1A	179.4 (2)
N1A—Ni1A—O1MA—C1MA	−53.4 (2)	C7A—C1A—C2A—O1A	2.3 (4)
O1A—Ni1A—O1MA—C1MA	38.3 (2)	C6A—C1A—C2A—C3A	0.7 (4)
O1AA—Ni1A—O1MA—C1MA	129.1 (2)	C7A—C1A—C2A—C3A	−176.5 (2)
N2A—Ni1A—O1MA—C1MA	−137.3 (2)	O1A—C2A—C3A—C4A	−179.2 (2)
O1B—Ni1B—O1MB—C1MB	−27.0 (2)	C1A—C2A—C3A—C4A	−0.4 (4)
N1B—Ni1B—O1MB—C1MB	64.4 (2)	C2A—C3A—C4A—C5A	−0.5 (4)
O1AA—Ni1B—O1MB—C1MB	−121.2 (2)	C3A—C4A—C5A—C6A	1.2 (4)
N2B—Ni1B—O1MB—C1MB	147.5 (2)	C3A—C4A—C5A—Cl1A	−178.1 (2)
O1A—Ni1A—N1A—C7A	−1.7 (2)	C4A—C5A—C6A—C1A	−0.9 (4)
O1—Ni1A—N1A—C7A	−95.8 (2)	Cl1A—C5A—C6A—C1A	178.4 (2)
O1MA—Ni1A—N1A—C7A	89.0 (2)	C2A—C1A—C6A—C5A	0.0 (4)
N2A—Ni1A—N1A—C7A	175.1 (2)	C7A—C1A—C6A—C5A	177.3 (3)
O1A—Ni1A—N1A—C8A	−176.78 (19)	C8A—N1A—C7A—C1A	177.7 (3)
O1—Ni1A—N1A—C8A	89.1 (2)	Ni1A—N1A—C7A—C1A	2.8 (4)
O1MA—Ni1A—N1A—C8A	−86.2 (2)	C6A—C1A—C7A—N1A	179.9 (3)
N2A—Ni1A—N1A—C8A	0.00 (19)	C2A—C1A—C7A—N1A	−2.9 (4)
N1A—Ni1A—N2A—C7	100.69 (18)	C7A—N1A—C8A—C9A	−152.7 (2)
O1—Ni1A—N2A—C7	1.24 (17)	Ni1A—N1A—C8A—C9A	22.8 (3)
O1AA—Ni1A—N2A—C7	−77.75 (17)	C7—N2A—C9A—C8A	−80.8 (3)
O1MA—Ni1A—N2A—C7	−169.59 (17)	C8—N2A—C9A—C8A	164.5 (2)
N1A—Ni1A—N2A—C9A	−22.88 (17)	Ni1A—N2A—C9A—C8A	42.5 (2)
O1—Ni1A—N2A—C9A	−122.33 (16)	N1A—C8A—C9A—N2A	−45.0 (3)
O1AA—Ni1A—N2A—C9A	158.67 (16)	Ni1B—O1B—C2B—C1B	1.1 (4)
O1MA—Ni1A—N2A—C9A	66.84 (16)	Ni1B—O1B—C2B—C3B	−178.32 (18)
N1A—Ni1A—N2A—C8	−142.47 (18)	C6B—C1B—C2B—O1B	−176.3 (2)
O1—Ni1A—N2A—C8	118.07 (17)	C7B—C1B—C2B—O1B	2.0 (4)
O1AA—Ni1A—N2A—C8	39.08 (17)	C6B—C1B—C2B—C3B	3.1 (4)
O1MA—Ni1A—N2A—C8	−52.75 (17)	C7B—C1B—C2B—C3B	−178.6 (2)
O1B—Ni1B—N1B—C7B	7.6 (2)	O1B—C2B—C3B—C4B	177.4 (2)
O1—Ni1B—N1B—C7B	100.9 (2)	C1B—C2B—C3B—C4B	−2.0 (4)
O1MB—Ni1B—N1B—C7B	−82.8 (2)	C2B—C3B—C4B—C5B	−0.3 (4)
N2B—Ni1B—N1B—C7B	−172.3 (2)	C3B—C4B—C5B—C6B	1.5 (4)
O1B—Ni1B—N1B—C8B	−178.14 (18)	C3B—C4B—C5B—Cl1B	−177.9 (2)
O1—Ni1B—N1B—C8B	−84.90 (18)	C4B—C5B—C6B—C1B	−0.4 (4)
O1MB—Ni1B—N1B—C8B	91.49 (18)	Cl1B—C5B—C6B—C1B	179.1 (2)
N2B—Ni1B—N1B—C8B	1.93 (18)	C2B—C1B—C6B—C5B	−2.0 (4)
N1B—Ni1B—N2B—C7	−101.07 (17)	C7B—C1B—C6B—C5B	179.6 (3)
O1—Ni1B—N2B—C7	−1.00 (17)	C8B—N1B—C7B—C1B	178.7 (3)
O1AA—Ni1B—N2B—C7	78.85 (17)	Ni1B—N1B—C7B—C1B	−7.3 (4)
O1MB—Ni1B—N2B—C7	170.79 (17)	C2B—C1B—C7B—N1B	1.5 (5)
N1B—Ni1B—N2B—C9B	21.94 (16)	C6B—C1B—C7B—N1B	179.8 (3)
O1—Ni1B—N2B—C9B	122.00 (16)	C7B—N1B—C8B—C9B	148.9 (2)
O1AA—Ni1B—N2B—C9B	−158.15 (16)	Ni1B—N1B—C8B—C9B	−25.7 (3)
O1MB—Ni1B—N2B—C9B	−66.21 (16)	C7—N2B—C9B—C8B	81.2 (3)
N1B—Ni1B—N2B—C9	141.20 (17)	C9—N2B—C9B—C8B	−164.4 (2)
O1—Ni1B—N2B—C9	−118.74 (17)	Ni1B—N2B—C9B—C8B	−43.0 (2)
O1AA—Ni1B—N2B—C9	−38.89 (17)	N1B—C8B—C9B—N2B	47.6 (3)
O1MB—Ni1B—N2B—C9	53.05 (16)	Ni1B—O1AA—C1AA—O2AA	−156.4 (2)
Ni1B—O1—C1—C2	−123.7 (2)	Ni1A—O1AA—C1AA—O2AA	34.0 (4)
Ni1A—O1—C1—C2	122.5 (2)	Ni1B—O1AA—C1AA—C2AA	25.8 (4)
Ni1B—O1—C1—C6	57.5 (3)	Ni1A—O1AA—C1AA—C2AA	−143.8 (2)

### Hydrogen-bond geometry (Å, °)

**Table:**

*D*—H···*A*	*D*—H	H···*A*	*D*···*A*	*D*—H···*A*
O1MA—H1MK···O2AA	0.84	1.77	2.586 (3)	162.
O1MA—H1MK···O1AA	0.84	2.66	3.029 (2)	108.
O1W—H1W1···O1A	0.82 (2)	1.86 (2)	2.679 (3)	175 (4)
O1W—H1W2···O1B	0.81 (2)	1.91 (2)	2.708 (3)	174 (3)
O1M—H1M···O1W^i^	0.84	1.74	2.577 (3)	170.
O1MB—H1MJ···O1M	0.84	1.83	2.658 (3)	167.
C6A—H6AA···O2AA^ii^	0.95	2.37	3.237 (4)	152.
C7A—H7AA···O2AA^ii^	0.95	2.32	3.211 (3)	156.

Symmetry codes: (i) *x*+1/2, −*y*+3/2, *z*; (ii) −*x*, −*y*+2, *z*+1/2.

## References

[bb1] Bruker (2000). *SMART* and *SAINT-Plus* Bruker AXS Inc., Madison, Wisconsin, USA.

[bb2] Flack, H. D. (1983). *Acta Cryst.* A**39**, 876–881.

[bb3] Fondo, M., Garcia-Deibe, A. M., Corbella, M., Ruiz, E., Tercero, J., Sanmartin, J. & Bermejo, M. R. (2005). *Inorg. Chem.* **44**, 5011–5020. 10.1021/ic0482741 15998029

[bb4] Fondo, M., Garcia-Deibe, A. M., Ocampo, N., Sanmartin, J. & Bermejo, M. R. (2007). *Dalton Trans.* pp. 414–416. 10.1039/b617374h 17213925

[bb5] Fondo, M., Garcia-Deibe, A. M., Ocampo, N., Sanmartin, J., Bermejo, M. R. & Llamas-Saiz, A. L. (2006). *Dalton Trans.* pp. 4260–4270. 10.1039/b606414k 16932819

[bb6] Fondo, M., Ocampo, N., Garcia-Deibe, A. M., Ruiz, E., Tercero, J. & Sanmartin, J. (2009). *Inorg. Chem.* **48**, 9861–9873. 10.1021/ic9014916 19761206

[bb7] Fondo, M., Ocampo, N., Garcia-Deibe, A. M., Vicente, R., Corbella, M., Bermejo, M. R. & Sanmartin, J. (2006). *Inorg. Chem.* **45**, 255–262. 10.1021/ic051194s 16390063

[bb8] Lu, L.-P., Lu, X.-P. & Zhu, M.-L. (2007). *Acta Cryst.* C**63**, m374–m376. 10.1107/S0108270107023256 17675688

[bb9] Paital, A. R., Ribas, J., Barrios, L. A., Aromi, G. & Ray, D. (2009). *Dalton Trans.* pp. 256–258. 10.1039/b811788h 19089004

[bb10] Paital, A. R., Wong, W. T., Aromi, G. & Ray, D. (2007). *Inorg. Chem.* **46**, 5727–5733. 10.1021/ic700496c 17569529

[bb11] Sheldrick, G. M. (2003). *SADABS* University of Göttingen, Germany.

[bb12] Sheldrick, G. M. (2008). *Acta Cryst.* A**64**, 112–122. 10.1107/S0108767307043930 18156677

